# CD109 exhibits a dynamic expression pattern in coronary endothelium and endocardial-derived valve mesenchyme during heart development with preserved morphogenesis following endothelial-specific deletion

**DOI:** 10.3389/fcell.2026.1867997

**Published:** 2026-06-19

**Authors:** Andrew B. Harvey, Jenna R. Drummond, Hannah G. Tarolli, Renélyn A. Wolters, Raymond N. Deepe, Inara Devji, Jeremy L. Barth, Robin Muise-Helmericks, Paula S. Ramos, Russell A. Norris, Andy Wessels

**Affiliations:** 1 Department of Regenerative Medicine and Cell Biology, College of Medicine, Medical University of South Carolina, Charleston, SC, United States; 2 Department of Medicine, Medical University of South Carolina, Charleston, SC, United States; 3 Department of Medicine, Emory University School of Medicine, Atlanta, GA, United States

**Keywords:** CD109, coronary vasculature, endothelial biology, heart development, heart valves, lineage tracing

## Abstract

**Background:**

*CD109* encodes a GPI-linked glycoprotein that acts as a signaling modulator in the TGF-β pathway. *CD109* has emerged in several genome-wide association studies as linked to coronary artery disease, myocardial infarction, and angina pectoris. Heterozygous loss-of-function mutations in *CD109* have also been reported in patients with congenital heart defects, suggesting potential developmental relevance, though *CD109* has never been investigated in the context of cardiovascular development. We previously identified *Cd109* upregulation in murine atrioventricular valves undergoing myxomatous degeneration following a reduction of epicardial-derived cells. Here, we characterize *Cd109* expression in the murine cardiovascular system and assess its function during development using *in vitro* and *in vivo* approaches.

**Results:**

We found that *Cd109* is strongly expressed in the endothelium of the coronary vasculature and in endocardial-derived subpopulations in the atrioventricular valves. This expression persists through key stages in cardiovascular development. Western blotting and immunostaining confirm endothelial expression in heart and lung tissues. siRNA-mediated knockdown of CD109 in primary human endothelial cells led to dysregulation of vascular development pathways and decreased tube formation capacity. We generated endothelial-specific *Cd109* knockout mice, eliminating *Cd109* expression from heart and lung tissues without overt consequences for atrioventricular valve or coronary vascular morphogenesis during heart development.

**Conclusion:**

CD109 exhibits a highly dynamic spatiotemporal expression pattern during cardiovascular development, with enriched expression in coronary endothelial cells and endocardial-derived subpopulations in the valves. Despite this striking developmental expression pattern, previously reported human genetic associations with cardiovascular diseases, and endothelial-associated phenotypes following siRNA-mediated CD109 knockdown in a primary human endothelial cell line, endothelial/endocardial-lineage deletion of *Cd109* did not produce overt abnormalities in atrioventricular valve or coronary vascular morphogenesis during embryonic development. Collectively, these findings identify CD109 as a useful marker of coronary endothelial and endocardial-derived valve cell populations and suggest that CD109 may function in a context-dependent or modulatory manner rather than as an essential regulator of cardiovascular morphogenesis under normal developmental conditions.

## 1 Introduction

### 1.1 Background and rationale for investigating CD109 in cardiovascular development

The cluster of differentiation 109 (*CD109*) gene encodes a glycosylphosphatidylinositol (GPI)-anchored cell surface glycoprotein expressed in a variety of cell types including many immune cells, endothelial cells, and human cancers (Sutherland et al., 1991; Murray et al., 1999; Batal et al., 2024; Hashimoto et al., 2004; Hasegawa et al., 2008; Sato et al., 2007; Ye et al., 2016). Previous work in cancer and epidermal biology shows that CD109 modulates several signaling pathways involved in cell differentiation and extracellular matrix (ECM) homeostasis, most notably through negative regulation of Transforming Growth Factor-Beta (TGF-β) signaling via interactions with both ligand and receptor components (Finnson et al., 2006; Tam et al., 2003; Tam et al., 2001; Man et al., 2012; Zhang et al., 2015). Additional studies have implicated CD109 in modulation of Hippo and EGFR signaling in various cancer contexts (Zhang et al., 2015; Lee et al., 2020a; Zhou et al., 2022; Mo et al., 2020; Lee et al., 2020b). While these findings suggest that CD109 influences processes important for tissue development and remodeling, its expression pattern and functional role in the heart have not yet been investigated. Global deletion of CD109 in mice has been reported to cause epidermal hyperplasia and transient impairment of hair growth (Mii et al., 2012). These CD109-deficient mice are viable, and do not display apparent abnormalities in other tissues (Mii et al., 2012).

Our interest in CD109 arose from RNA-seq analysis in a mouse model of myxomatous mitral valve disease (*Wt1* ^Cre^;*Sox9* ^fl/fl^), where *Cd109* was significantly upregulated in atrioventricular valve leaflets during valve disease progression (Harvey et al., 2023). Follow up expression studies showed that CD109 is present in a population of mesenchymal cells at the distal ends of developing valve leaflets, and its expression expands in the diseased state (Harvey et al., 2023). We also observed CD109 expression in the vascular endothelium of the developing coronary vessels (Harvey et al., 2023). These observations, together with its reported signaling functions in other systems, led us to investigate CD109 expression and its potential role in cardiovascular development.

During cardiovascular development, endocardial cells undergo endothelial-to-mesenchymal transformation (EndMT) to form the atrioventricular (AV) cushions, which subsequently elongate and remodel into mature valve leaflets (Mercado-Pimentel and Runyan, 2007; Inai et al., 2008; Markwald et al., 1977; Goddard et al., 2017; Hogers et al., 1999; Hove et al., 2003). At a similar timepoint, coronary vessels arise beginning around murine embryonic day (E)10.5 from the sinus venosus, forming a subepicardial vascular plexus that expands across the heart and invades the underlying myocardium to provide oxygenated blood to the heart muscle (Chen et al., 2014; Pampols-Perez et al., 2025; Wu et al., 2013; Dyer et al., 2014). These processes involve coordinated contributions from endothelial, mesenchymal, and epicardial cell populations and are regulated by tightly controlled signaling interactions (Brade et al., 2013; Muñ et al., 2010). This context provides a framework for our investigations of CD109 expression and function in the heart.

### 1.2 Genetic evidence of a role of CD109 in the heart

Recent human genetic association studies provide additional, though indirect, evidence that CD109 may be relevant in cardiovascular biology. Genome-wide association studies (GWAS)—accessed via the NHGRI-EBI GWAS catalog—have identified variants at the *CD109* locus associated with coronary artery disease (CAD), myocardial infarction (MI), and angina pectoris (chest pain). Koyama et al. found that an intronic variant in *CD109* (rs56171536) was positively associated with CAD in a large cohort from the Biobank of Japan (Koyama et al., 2020), and Sakaue et al. reported associations between variants in the same locus and both MI and angina pectoris across a broad phenotypic screen (Sakaue et al., 2021). In addition to these common variant associations, rare coding variants in *CD109* have been reported in patients with congenital heart disease (CHD). Jin et al. identified six individuals harboring heterozygous loss-of-function (LOF) variants in CD109 through whole exome sequencing (WES) of 2,871 CHD probands and their parents (Jin et al., 2017). These patients presented with a range of diagnosed structural cardiovascular defects listed in Table 1. However, in each case, additional LOF variants in other genes were also present, making causality difficult to attribute. While these studies suggest that CD109 may contribute to cardiovascular phenotypes, direct functional evidence of a role for CD109 in cardiovascular development is lacking.

**TABLE 1:** Human congenital heart disease patients with heterozygous loss-of-function variants in CD109.

Blinded ID	CD109 Variant position (CHR 6)	REF	ALT	Variant location	Cause	CD109 AA Change	All LOF gene variants	CHD
1–02788	74468691	T	A	Exonic	Stopgain	p.L233X	*ADAMTS4, APOA1BP, C7orf61, * ***CD109*** , NLRC3, TRPM5, MINK1*	Tetralogy of fallot
1–00924	74466386	ATTTCAGG	A	Exonic	Frameshift Deletion	p.F219fs	***CD109*** *, ATP8A2*	Aortic stenosis - valvar | atrial septal defect, secundum | hypoplastic aortic annulus | hypoplastic aortic isthmus | hypoplastic left heart syndrome | hypoplastic left ventricle (subnormal cavity volume) | mitral stenosis | ventricular septal defect, membranous | ventricular septal defect, multiple | ventricular septal defect, muscular
1–01242	74519731	C	G	Exonic	Stopgain	p.S1127X	MYO15A**, ***CD109*** *, HRAS, PLB1, PLD1, PTPRJ, SLC22A11, STRA13, TMEM52B, VWA3B, WDR11*	Absence of the suprarenal inferior vena cava with azygous continuation | atrial inversion (situs inversus of the atria) | common atrium | discontinuity of pulmonary arteries | heterotaxy | left superior vena cava entering left atrium | pulmonary stenosis, subvalvar | pulmonary stenosis, valvar | single ventricle with unbalanced atrioventricular canal defect
1–05534	74492480	T	C	Splicing	.	.	***CD109*** , GBGT1, SIGLEC8, SREK1IP1, IL31RA*, KNDC1*, TBX18*	Atrial septal defect | partially anomalous pulmonary veins | sinus venosus septal defect, superior type
1–04009	74466406	G	GTT	Splicing	.	.	***CD109*** *, CPNE3, ELP2, EPPK1, PCDH12, SMCO2*	Patent ductus arteriosus | ventricular septal defect, muscular
1–12480	74519820	C	T	Exonic	Stopgain	p.Q1157X	*ADA, ATP13A5, * ***CD109*** , DHDDS, HFE2, KMT2D*, PI4KA*	Atrial septal defect, secundum | hypoplastic left heart syndrome

* Indicates a *de novo* pathogenic variant; ** indicates compound heterozygous pathogenic variant.

Whole exome sequencing data from Jin et al. identifying six individuals with CHD harboring loss-of-function (LOF) variants in CD109. Genomic position (Chr6), reference and alternate alleles, variant classification, predicted amino acid change, and co-occurring LOF variants in other genes are shown. Reported cardiovascular phenotypes are listed as described in the original study.

### 1.3 Overview of experimental design

Given the absence of detailed information regarding CD109 in the heart, we sought to define its spatiotemporal expression during cardiovascular development and to assess whether it is required for normal heart morphogenesis. We performed a systematic analysis of CD109 expression across key stages of murine heart development, focusing on the valves and coronary vasculature. To evaluate potential endothelial functions, we employed siRNA-mediated knockdown in primary human endothelial cells to evaluate transcriptional changes and angiogenic behavior *in vitro*. Finally, we generated an endothelial/endocardial lineage-specific conditional knockout mouse to test whether CD109 is required for normal cardiovascular development *in vivo*. Together, this study establishes a foundational characterization of CD109 expression in the developing cardiovascular system and provides an initial assessment of its functional requirement in this context.

## 2 Results

### 2.1 CD109 expression during development

**FIGURE 1:**
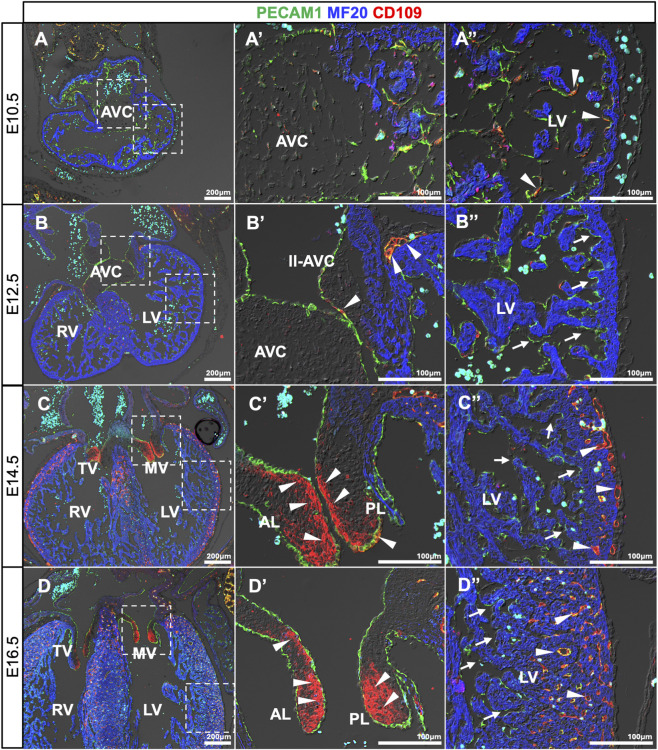
CD109 expression in cardiovascular development. **(A–D’’)** Immunofluorescent staining for CD109 (red), PECAM1 to mark endothelial cells (green), and MF20 to mark myocardium (blue). Boxed areas are magnified in **(A’–D’’)**. **(A,B,C,D)** Four-chambered transverse view of developing heart at indicated embryonic timepoints. **(A’,B’,C’,D’)** Magnified images of boxed areas showing CD109 expression in developing mitral valve. Arrowheads point to CD109^+^ populations, primarily in the distal, flow-facing regions of the valve leaflets. **(A’’,B’’,C’’,D’’)** Magnified images of boxed areas showing CD109 expression in ventricular wall. Arrowheads point to CD109+ coronary endothelial cells in the ventricular wall and subepicardium. Arrows point to CD109^–^ endocardium. A minimum of n = 3 embryos per time point were examined. (Abbreviations: AL, anterior leaflet; AVC, atrioventricular cushion; ll-AVC, left lateral atrioventricular cushion; LV, left ventricle; MV, mitral valve; PL, parietal leaflet; TV, tricuspid valve).

To determine the spatiotemporal expression pattern of CD109 in relation to cardiac valve and coronary system development, we conducted immunostainings on wildtype embryos in a developmental series at E10.5, E12.5, E14.5, and E16.5. Sections were immunolabeled with antibodies for CD109, PECAM1 to mark endothelial cells, and MF20 to mark myocardium. CD109 expression is first detected early in heart development at E10.5 in the endocardial cells lining the ventricles (Figures 1A-A”). There is minimal detectable CD109 signal in the cushion mesenchyme or cushion endothelium at this timepoint (Figure 1A’). At E12.5, CD109 expression in the endocardium itself is diminished (Figures 1B–B”). However, we begin to see expression in the endocardial lining of the AV cushions, as well as in the endothelial cells of the subepicardial coronary vessels in the atrioventricular sulci (Figure 1B’). By E14.5, we see a robust population of mesenchymal cells in the developing AV valve leaflets that expresses CD109, particularly on the flow-facing region (also called the shear side) of the leaflets (Figures 1C–C’). A small portion of the endocardial lining of the leaflets at the distal tip stains positive for both CD109 and PECAM1. There is widespread expression of CD109 in the coronary vascular plexus that has spread throughout the subepicardial space and within the ventricular myocardium, but we no longer detect CD109 expression in the endocardium proper (Figure 1C”). This expression pattern is maintained at E16.5 as CD109 can be seen both in the endothelial cells of the coronary vasculature, as well as in the distal regions of the developing AV valve leaflets, but not in the endocardium proper (Figures 1D–D”).

**FIGURE 2:**
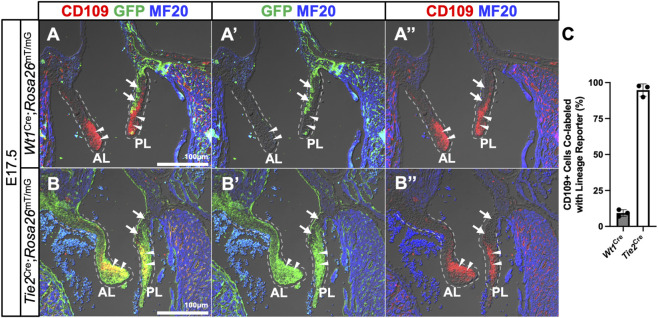
CD109 is preferentially expressed in endocardial-derived mesenchyme of the AV valves. Immunolabeled images of mitral valve in E17.5 specimens with lineage trace reporters. The epicardial [Wt1^Cre^ in **(A–A”)**] and endocardial [Tie2^Cre^ in **(B–B”)**] lineages are marked by GFP reporter expression (green). CD109 (red) labels a subpopulation of valve mesenchyme in the distal region of the leaflets, as well as coronary endothelial cells in the ventricular wall and interventricular septum. For clearer visualization, **(A’)** and **(B’)** show GFP channel without CD109, and **(A’’)** and **(B’’)** show CD109 channel without GFP. MF20 (blue) marks myocardium. Arrows indicate CD109^–^ regions of epicardial-derived cell contribution to the parietal leaflet, while arrowheads indicate endocardial-derived cells that are co-labeled with CD109 expression. Note that not all endocardial-derived cells express CD109, but virtually all CD109^+^ cells are endocardial-derived. **(C)** Quantification of the percentage of CD109^+^ cells in the parietal leaflet of the mitral valve that are co-labeled with GFP lineage reporter in the epicardial lineage (*Wt1* ^Cre^, n = 3, 9.3% ± 2.3%) and endocardial lineage (*Tie2* ^Cre^, n = 3, 94.8% ± 4.3%) (Abbreviations: AL, anterior leaflet; PL, parietal leaflet).

During AV valve development, an invasive population of epicardial-derived cells (EPDCs) migrate through the AV junctional myocardium and into the endocardial-derived lateral (or parietal) AV cushion (Wessels et al., 2012; Gittenberger-de Groot et al., 1998). These EPDCs eventually make up over 50% of the cells in the parietal leaflets postnatally, though few EPDCs are found in the anterior or septal-associated leaflets, largely composed of cells derived from endocardial-to-mesenchymal transformation (EndMT) (Harvey et al., 2023; Lockhart et al., 2014). We previously reported an expanded population of CD109-expressing mesenchymal cells in two mouse models in which reduced EPDC contribution to the parietal leaflets of the AV valves during development results in valve abnormalities postnatally (*Wt1* ^Cre^;*Sox9* ^fl/fl^ and *Wt1* ^Cre^;*Alk3* ^fl/fl^) (Harvey et al., 2023). Thus, we were interested in the lineage origin of CD109-expressing cells within the developing AV valves. To explore this, we used the Cre-loxP system in combination with a Rosa26 reporter allele, in which Cre recombinase expressed in specific cell populations permanently activates GFP expression, allowing those cells and their descendants to be identified by immunofluorescence. *Wt1* ^Cre^;*Rosa26* ^mT/mG^ embryos were used to label EPDCs (Wessels et al., 2012), and *Tie2* ^Cre^;*Rosa26* ^mT/mG^ embryos were used to label the endocardial/endothelial lineage (Kisanuki et al., 2001). In *Wt1* ^Cre^;*Rosa26* ^mT/mG^ lineage-trace specimens, the majority of EPDCs (marked by GFP) within the developing valve leaflets were CD109-negative (arrows) with rare overlap observed between EPDCs and CD109 (Figures 2A–A”). By contrast, in *Tie2* ^Cre^;*Rosa26* ^mT/mG^ specimens, most CD109^+^ cells (arrowheads) within the valve leaflets were also GFP^+^, indicating their endocardial origin (Figures 2B–B”). Quantification revealed that less than 10% of CD109^+^ cells within the parietal leaflet were co-labeled with the *Wt1* ^Cre^ lineage reporter, while 95% of CD109^+^ cells within the parietal leaflet were co-labeled with the *Tie2* ^Cre^ lineage reporter (Figure 2C). EPDCs do not contribute to the anterior leaflet. Thus, as expected, 100% of the CD109^+^ cells quantified in the anterior leaflet were labeled with the *Tie2* ^Cre^ lineage reporter. In the *Tie2* ^Cre^;*Rosa26* ^mT/mG^ specimens, the GFP^–^cells in the parietal leaflet (arrows) are also CD109^–^ (Figures 2B–B”). Not all endocardial-derived cells expressed CD109, indicating that CD109 marks only a subset of endocardial-derived cells and not the entire population. However, CD109 is interestingly the only identified marker, to our knowledge, that preferentially labels mesenchyme of endocardial origin in the developing parietal AV valve leaflets, and very few EPDCs. Together, these data indicate that CD109 expression in valve development preferentially marks a subset of endocardial-derived mesenchymal cells in the distal, flow-facing regions of the valve leaflets.

**FIGURE 3:**
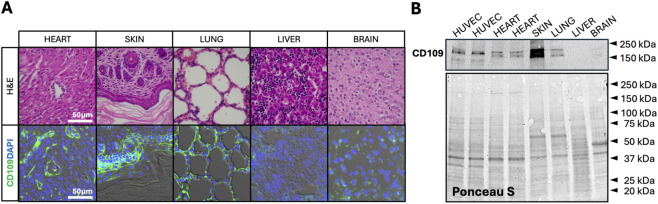
CD109 expression in neonatal organs. **(A)** Hematoxylin and Eosin staining of neonatal organs, paired with immunofluorescent labeling of CD109 (green) in the same tissues. DAPI counterstain to show nuclei in blue. **(B)** Western blot for CD109 in primary human umbilical vein endothelial cells (HUVECs) in addition to other organs. Ponceau S total protein stain shows comparable protein loading in each lane. CD109 is detected in HUVECs, heart, skin, and lung tissues, but minimal detection in liver or brain.

To determine which other tissues and cell types express CD109, we analyzed neonatal mouse organs by immunofluorescent labeling and Western blot. These analyses revealed strong CD109 expression in heart, skin, and lung tissues, but very little CD109 in liver or brain (Figures 3A,B). We also detected CD109 expression in primary human umbilical vein endothelial cells (HUVECs) by Western blot, supporting their suitability as an *in vitro* culture system for analyzing CD109 function in endothelial cells (Figure 3B).

### 2.2 Knockdown of CD109 alters angiogenic programs and tube formation *in vitro*

**FIGURE 4:**
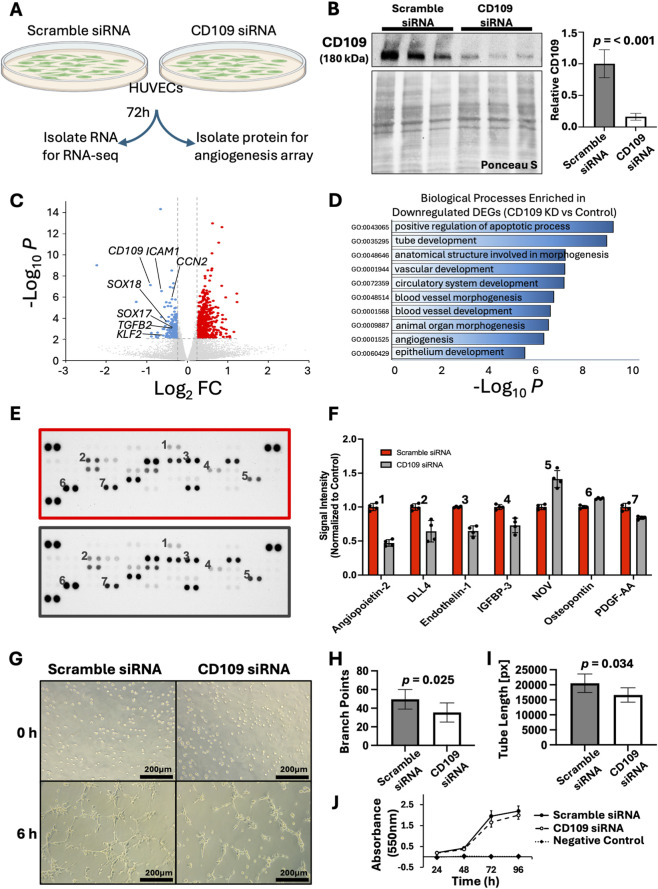
CD109 knockdown alters endothelial cell programs and tube formation *in vitro*. **(A)** Depiction of experimental approach for siRNA mediated knockdown of CD109 in primary HUVECs. **(B)** Western blot confirms that 72 h treatment with siRNA targeting CD109 results in approximately 85% knockdown of CD109 protein (n = 4 independent experiments). **(C)** Volcano plot of differentially expressed genes identified via bulk RNA-seq from n = 3 control versus n = 3 CD109 knockdown HUVEC cultures. **(D)** Gene Ontology analysis of downregulated genes in CD109 knockdown HUVECs indicates dysregulation of biological processes associated with vasculature development. **(E)** Human Angiogenesis Array profiling of protein isolated from control (red) versus CD109 knockdown (gray) HUVECs revealed differential expression of seven angiogenesis-related proteins. Pixel intensities relative to control blots are quantified in **(F)**. **(G)** Tube formation assay of control (left column) versus CD109 knockdown (right column) HUVECs seeded on growth factor-reduced Matrigel. **(H,I)** Quantification of the number of branch points **(H)** and total tube length **(I)** per field indicated a decreased tube formation propensity after CD109 knockdown (n = 6 wells per experimental condition, 2 independent experiments). **(J)** Crystal Violet staining of adherent HUVECs to assay proliferation showed no differences between scramble control treated versus CD109 knockdown cultures over 4 days (n = 4 wells per timepoint). Negative control measurements were taken from wells with no cells seeded.

In an effort to elucidate the potential role of CD109 in the coronary endothelium, we next performed a series of experiments to test the consequence of CD109 depletion in primary endothelial cells. HUVECs were used as a tractable primary culture system for identifying endothelial-associated responses to CD109 perturbation, having identified CD109 expression in HUVECs by Western blot, though they are not coronary or cardiac-specific endothelial cells by nature. CD109 expression was reduced in HUVECs via siRNA-mediated knockdown. Seventy-two hours after transfection with siRNA targeting CD109 or scramble control siRNA, total RNA and total protein were harvested for transcriptomic and protein analyses (Figure 4A). Western blot analysis confirmed approximately 85% reduction in CD109 protein levels in knockdown cultures compared to controls 72 h post-transfection (Figure 4B). Bulk RNA-seq revealed 285 significantly downregulated genes in CD109 knockdown cultures compared to controls, and 588 significantly upregulated genes (FDR P_adj_<0.1, Log_2_FC>0.2) (Figure 4C; Supplementary Table S1). Given the exploratory nature of this transcriptional profiling, these thresholds were employed to capture broader pathway-level trends in downstream analysis. Functional enrichment analysis revealed limited biological process enrichment in upregulated genes, with “RNA splicing” (GO:0008380) being the only significantly enriched parent term. However, downregulated genes were enriched for multiple endothelial processes including “tube development,” “vascular development,” “blood vessel morphogenesis,” and “angiogenesis,” among others (Figure 4D; Supplementary Table S2). Genes downregulated in these vasculature related terms included *TGFB2*, *SOX18*, *SOX17*, *CCN2*, *KLF2*, and *ICAM1*, which have established roles in vascular development and endothelial function (Supplementary Table S2).

To determine whether loss of CD109 affected angiogenesis-related protein expression, we performed a human angiogenesis proteomic array on protein isolated from control and CD109 knockdown HUVECs 72 h post-transfection (Figure 4E). 7proteins out of the 55 probed were differentially expressed following CD109 knockdown (Figure 4F; Supplementary Table S3). The most downregulated proteins included Angiopoietin-2 (ANG-2), Delta-like Notch ligand 4 (DLL4), and Endothelin-1 (ET-1), with each reduced approximately two-fold. None of the differentially expressed proteins overlapped with RNA-seq DEGs, suggesting that some effects of CD109 knockdown occur independently of transcriptional regulation.

To functionally investigate the role of CD109 in the angiogenic behavior of endothelial cells, we performed an *in vitro* tube formation assay with control and CD109-knockdown HUVECs seeded on growth factor-reduced Matrigel (Figure 4G). Six hours after seeding, CD109-knockdown cells showed reduced angiogenic capacity as quantified by total tube length and number of branch points when compared to control cultures (Figures 4H,I). No significant difference in proliferation was observed between control and CD109-knockdown cultures assessed via crystal violet staining (Figure 4J).

### 2.3 Endothelial-specific knockout of Cd109 does not alter valve or vascular morphogenesis *in vivo*

Given its expression in cells of the endothelial/endocardial lineage, we sought to determine whether CD109 plays an important role in these populations during cardiovascular development *in vivo*. Our *in vitro* analyses also suggested CD109 may influence endothelial gene programs and angiogenic behavior. We generated endothelial-specific *Cd109* knockout mice to determine whether CD109 is required for valve or vascular morphogenesis. Since the semilunar valves receive substantial contributions from the cardiac neural crest and second heart field lineages, we focused our analysis on the AV valves which are predominantly endocardial-derived, with additional contributions from the epicardial lineage to the parietal leaflets later in development.

**FIGURE 5:**
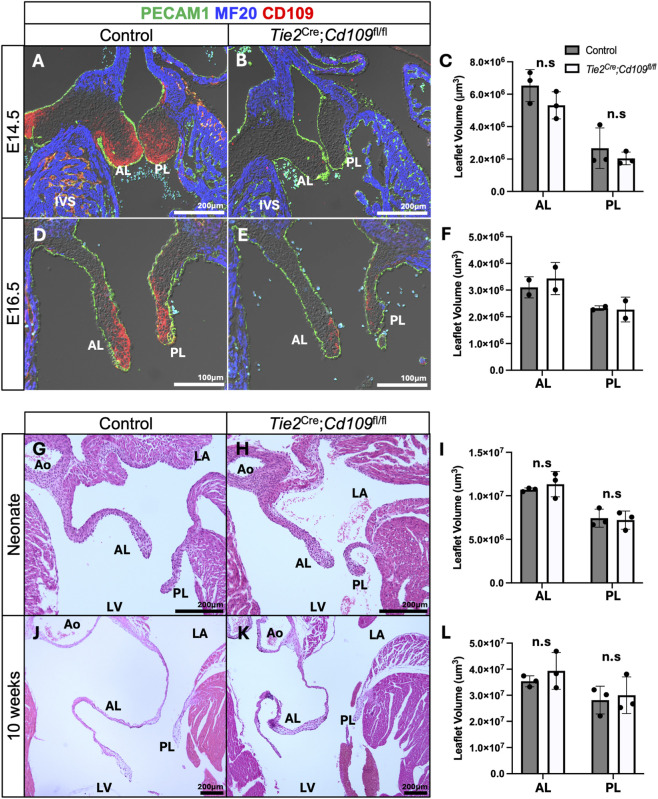
Loss of *Cd109* in the endothelial lineage does not affect atrioventricular valve morphogenesis. **(A–F)** Immunofluorescent labeling of CD109 (red), PECAM1 (green), and MF20 (blue) in E14.5 **(A,B)** and E16.5 **(D,E)** control and *Tie2* ^Cre^;*Cd109* ^fl/fl^ specimens indicates CD109 expression in valve mesenchyme in the distal components of the mitral valve leaflets in controls **(A,D)** with efficient CD109 knockout in *Tie2* ^Cre^;*Cd109* ^fl/fl^ specimens **(B,E)**. Quantification of anterior and parietal mitral valve leaflet volumes at E14.5 **(C)** revealed no significant differences between genotypes (two-tailed unpaired Student’s t-test; anterior leaflet *p* = 0.179, parietal leaflet *p* = 0.890; n = 3 embryos/genotype). Similar leaflet volumes were observed at E16.5 **(F)**, although interpretation at this stage is limited by sample size (n = 2 embryos per genotype). **(G–L)** Hematoxylin and Eosin stains of postnatal mitral valve tissue in neonatal **(G,H)** and 10-week **(J,K)** control and *Tie2* ^Cre^;*Cd109* ^fl/fl^ specimens show no differences between genotypes in valve morphology. Quantifications revealed no significant differences between genotypes (two-tailed unpaired Student’s t-test; neonatal anterior leaflet *p* = 0.498, parietal leaflet *p* = 0.811; 10-week anterior leaflet *p* = 0.401, parietal leaflet *p* = 0.729; n = 3 animals per genotype for each timepoint). (Abbreviations: AL, anterior leaflet; Ao, aorta; LA, left atrium; LV, left ventricle; n. s., not significant; PL, parietal leaflet).

The previously described Tie2-Cre model (Kisanuki et al., 2001) was used to generate *Tie2* ^Cre^;*Cd109* ^fl/fl^ mice in which *Cd109* is permanently deleted from endothelial cells and their progeny. *Tie2* ^Cre^ additionally labels some hematopoietic lineages (Tang et al., 2010), and thus does not represent a strictly coronary endothelial- or endocardial-specific deletion strategy, but it is widely used for generation of endothelial knockouts. We examined the structure of the AV valves in control and conditional knockout specimens to assess whether CD109 is required for normal valve development. As previously mentioned, the AV valves arise primarily from endocardial-to-mesenchymal transformation (EndMT), such that Cre-mediated recombination in this model targets most of the cells in the early valve structures. Beginning around E14.5, however, epicardial-derived cells (EPDCs) migrating through the atrioventricular junction will also contribute to the parietal leaflets. Immunostaining confirmed effective deletion of CD109 in the valve mesenchyme of *Tie2* ^Cre^;*Cd109* ^fl/fl^ specimens (Figures 5A,B). Immunofluorescent and histological analyses of the mitral valve at E14.5 (Figures 5A–C), E16.5 (Figures 5D–F), neonatal (Figures 5G–I), and 10-week (Figures 5J–L) stages revealed no overt morphological differences between control and *Tie2* ^Cre^;*Cd109* ^fl/fl^ valves. Valve leaflet structure and organization appeared to be the same across all stages examined. Quantification of anterior and posterior leaflet volumes showed no significant differences between genotypes at any time point (Figures 5C,F,I,L). While all quantitative analyses were performed on the mitral valve, no morphological differences across genotype were observed by qualitative examination of the tricuspid valve. Based on these observations, it appears that CD109 in the endothelial/endocardial lineage is not critically important for normal valve development.

We next examined coronary vascular development in control and *Tie2* ^Cre^;*Cd109* ^fl/fl^ hearts. The coronary system is the heart’s own vascular network that develops mid-gestation to provide for the increased metabolic needs of the growing heart. Endothelial cells derived mainly from the sinus venosus give rise to a subepicardial vascular plexus that spreads across the heart in response to VEGF signaling from the epicardium, invades into the underlying myocardium, and remodels throughout development into a mature vascular network (Chen et al., 2014; Dyer et al., 2014). Since CD109 is expressed in the coronary endothelium, we wanted to determine whether it plays an important role in this process during heart development.

**FIGURE 6:**
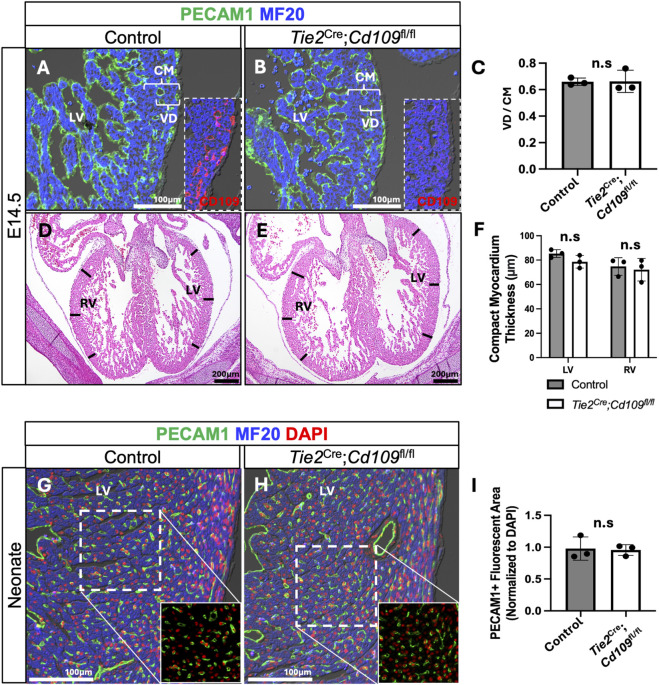
Loss of *Cd109* in the endothelial lineage does not affect coronary vascular development. **(A,B)** Immunofluorescent staining of E14.5 hearts showing coronary vasculature within the left ventricular wall labeled by PECAM1 (green) and MF20 (blue) to mark myocardium in control and *Tie2* ^Cre^;*Cd109* ^fl/fl^ specimens. Inset dotted boxes highlight CD109 expression (red), demonstrating efficient deletion of CD109 in *Tie2* ^Cre^;*Cd109* ^fl/fl^ hearts. **(C)** Quantification of the depth of vascular invasion (deepest PECAM1^+^ vessel) normalized to compact myocardium thickness (VD/CM) shows no significant difference between genotypes (two-tailed unpaired Student’s t-test; *p* = 0.948; n = 3 embryos per genotype). **(D,E)** Hematoxylin and Eosin staining of E14.5 hearts with black lines indicating regions where myocardial thickness measurements were taken. **(F)** Quantification of compact myocardial thickness in the LV and RV reveals no significant differences between control and *Tie2* ^Cre^;*Cd109* ^fl/fl^ specimens (two-tailed unpaired Student’s t-test; LV, *p* = 0.132; RV, *p* = 0.715; n = 3 embryos per genotype). **(G,H)** Immunofluorescent staining of neonatal hearts showing PECAM1 (green), myocardium (MF20, blue), and nuclei (DAPI, red). Insets show PECAM1 and DAPI channels split from the boxed regions which were used for vascular density quantification in cell profiler. **(I)** Quantification of vascular density as PECAM1^+^ area normalized to DAPI^+^ area indicating no significant difference between control and *Tie2* ^Cre^;*Cd109* ^fl/fl^ neonates (two-tailed unpaired Student’s t-test; *p* = 0.855; n = 3 embryos per genotype). (Abbreviations: CM, compact myocardium; LV, left ventricle; n. s., not significant; RV, right ventricle; VD, vascular depth).

At E14.5, PECAM1 immunolabeling shows similar vascular patterning in control and *Tie2* ^Cre^;*Cd109* ^fl/fl^ hearts, despite efficient knockout of CD109 in coronary endothelial cells (Figures 6A,B). To assess vascular invasion of the compact myocardium, we quantified the depth of PECAM1^+^ vessels relative to total thickness of the compact myocardium. This analysis revealed no significant differences in normalized vascular plexus depth between control and knockout embryos at E14.5 (Figure 6C). We then directly measured the thickness of the compact myocardium between control and *Tie2* ^Cre^;*Cd109* ^fl/fl^ hearts, which revealed no differences between genotypes (Figures 6D–F). Murine models with compromised coronary vascular development often exhibit reduced myocardial thickness, which may, in part, reflect insufficient vascular support for myocardial growth (Wu et al., 2013; Merki et al., 2005; Kwee et al., 1995). Histologically, the myocardial structure appeared identical between genotypes with respect to thickness and organization of the compact myocardium and trabecular network (Figures 6D,E). In neonatal specimens, we assessed coronary vascular density by quantifying PECAM1^+^ area normalized to DAPI^+^ nuclear area. This analysis similarly revealed no differences in vessel density within the ventricular myocardium between control and *Tie2* ^Cre^;*Cd109* ^fl/fl^ hearts (Figures 6G–I). We acknowledge that deletion of *Cd109* may have effects on parameters that were not investigated in the context of this study. However, based on these results, we conclude that endothelial-specific loss of *Cd109* does not grossly impair AV valve morphogenesis, coronary vascular morphogenesis, or myocardial growth during development.

**FIGURE 7:**
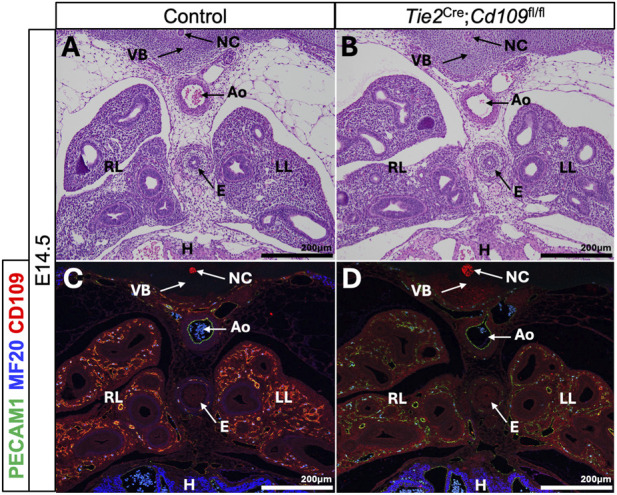
Loss of *Cd109* in the endothelial lineage does not affect lung development. **(A,B)** Hematoxylin and Eosin staining of lungs from control and *Tie2* ^Cre^;*Cd109* ^fl/fl^ specimens at E14.5 reveals comparable morphology between genotypes. Lobular organization and the distribution of bronchioles and surrounding architecture appear similar in control and knockout specimens. **(C,D)** Immunofluorescent staining for PECAM1 (green), CD109 (red), and MF20 (blue) demonstrates loss of CD109 expression in PECAM1^+^ endothelial cells in *Tie2* ^Cre^;*Cd109* ^fl/fl^ lungs compared to controls, confirming endothelial-specific deletion. The pattern and distribution of the pulmonary vasculature is unchanged despite loss of CD109 (n = 3 embryos per genotype). (Abbreviations: NC, notochord; VB, vertebral body; Ao, aorta; RL, right lung; LL, left lung; E, esophagus; H, heart).

Since CD109 expression was detected in the lungs (Figure 3), we next examined whether endothelial-specific deletion of *Cd109* affects lung development. Histological analysis by H&E revealed no overt differences in lung morphology between control and *Tie2* ^Cre^;*Cd109* ^fl/fl^ embryos (Figures 7A,B). The overall lobular architecture, as well as the distribution and organization of bronchioles and surrounding endothelial cells, appeared comparable between genotypes. Immunostaining at E14.5 confirmed loss of CD109 in PECAM1+ endothelial cells *Tie2* ^Cre^;*Cd109* ^fl/fl^ lungs compared to controls (Figures 7C,D). The same qualitative observations were made at additional developmental and postnatal stages. These findings indicate that endothelial CD109 is not required for gross pulmonary morphogenesis during development.

## 3 Discussion

In this study, we provide a spatiotemporal characterization of CD109 expression in the developing cardiovascular system and evaluate its functional role in the endothelial lineage both *in vitro* and *in vivo*. We find that CD109 exhibits a dynamic expression pattern during murine heart development, beginning with transient expression in early ventricular endocardium and later becoming enriched in coronary endothelial cells as the vascular plexus develops. CD109 also marks a subset of endocardial-derived mesenchymal cells localized to the distal, flow-facing regions of the atrioventricular valve leaflets. Lineage tracing demonstrates that these CD109^+^ mesenchymal cells arise predominantly from the endocardial lineage, with minimal expression in epicardial-derived cells. Analysis of additional tissues revealed strong CD109 expression in skin, consistent with previous studies (Mii et al., 2012; Vorstenbosch et al., 2017; Xu et al., 2026), as well as expression in lung endothelium, but minimal expression in liver or brain.

CD109 expression in the coronary vasculature makes it a useful marker for coronary endothelial cells during heart development, with greater specificity for vasculature than more broadly expressed pan-endothelial markers such as PECAM1. In the AV valves, CD109 is unique in its preferential labeling of endocardial-derived cells to the exclusion of EPDCs. It nonetheless marks only a subset of valve mesenchyme rather than the entire endothelial/endocardial lineage. This pattern is consistent with growing recognition of the heterogeneity among valve interstitial cell populations during valve development and postnatal remodeling (Horne et al., 2015; Hulin et al., 2019). CD109 is enriched in flow-exposed regions of the valve leaflets, a domain known to experience distinct mechanical and signaling environments that influence extracellular matrix organization (Stephens et al., 2008; Kodigepalli et al., 2020; Wang et al., 2023). Given its reported role as a modulator of TGF-β signaling (Man et al., 2012; Zhang et al., 2015; Winocour et al., 2014), CD109 expression in this region may reflect participation in local signaling environments influenced by biomechanical forces. However, direct testing of this possibility was not done in this study.

Our *in vitro* data demonstrated that knockdown of CD109 altered endothelial gene programs associated with vascular development and angiogenesis, including downregulation of *SOX17*, *SOX18*, *KLF2*, and TGFB2 expression (Zhou et al., 2015; Lange et al., 2014; Kim et al., 2023; Chen et al., 2019; Novodvorsky and Chico, 2014). The reduction in *KLF2* expression is notable given its established role as a central mediator of endothelial responses to shear stress in both vascular and valvular endothelium (Novodvorsky and Chico, 2014; Pace et al., 2025; Goddard et al., 2017). Interestingly, *CD109* was reported among 171 genes, including *KLF2*, that were upregulated in response to laminar flow conditions in two endothelial cell lines (Helle et al., 2020), raising the possibility that CD109 may intersect with flow-responsive signaling networks, consistent with its expression pattern. Functionally, CD109 knockdown reduced tube formation capacity without changes in proliferation, indicating an effect on angiogenic behavior rather than general cell viability. These findings are consistent with a prior report in which shRNA-mediated knockdown of CD109 in HUVECs negatively influenced tube formation capacity without affecting proliferation (Ye et al., 2016). Together, these data indicate that *CD109* can influence endothelial programs associated with angiogenic function *in vitro*.

Despite these *in vitro* effects and its distinct expression pattern *in vivo*, endothelial/endocardial-specific deletion of *Cd109 in vivo* did not result in overt defects in valve morphogenesis, coronary vascular development, or lung morphology in the developmental stages examined. *Tie2* ^Cre^;*Cd109* ^fl/fl^ mice exhibited loss of CD109 expression in the endothelial lineage but showed no significant differences in atrioventricular valve morphogenesis, coronary plexus formation, or myocardial growth across the developmental stages analyzed. While these findings suggest that CD109 in the endothelial lineage is not required for normal cardiovascular development, the morphology-based analyses and sample sizes in this study may not detect subtle, low penetrance, stress-dependent, or later onset phenotypes. These findings are consistent with the possibility that CD109 functions as a fine-tuning modulatory factor, rather than an essential component of signaling networks such as TGF-β, which is regulated by multiple overlapping ligands, receptors, and co-factors. This lack of a morphological phenotype following *Cd109* deletion may be a result of functional redundancy or compensatory mechanisms. CD109 may instead function in a more context-dependent manner, perhaps important in conditions where signaling balance is perturbed. This interpretation is supported by our prior observation that CD109-expressing valve mesenchymal populations expand in mouse models of myxomatous valve disease associated with reduced epicardial-derived cell contribution (Harvey et al., 2023). This context-dependent framework may help in interpreting human genetic studies linking *CD109* to coronary artery disease, myocardial infarction, and congenital heart defects (Koyama et al., 2020; Sakaue et al., 2021; Jin et al., 2017). While these associations implicate the *CD109* locus in cardiovascular phenotypes, they do not establish causality or define underlying mechanisms. Collectively, our findings suggest that CD109 in the endothelial lineage is not essential for normal cardiovascular development, but may contribute more prominently to modulating endothelial or valvular responses to other genetic or environmental stressors.

There are limitations due to the nature of this study that should be considered. *In vivo* analyses were limited to morphological endpoints and did not assess functional parameters such as endothelial responsiveness to flow or susceptibility to injury. In addition, *in vivo* deletion may permit compensation during development that limits the functional consequences seen by a more acute knockdown *in vitro*. While the HUVEC experiments provide a suitable system for identifying endothelial-associated responses to CD109 knockdown, they do not accurately recapitulate the biological context of developing coronary endothelial or endocardial-derived populations *in vivo*. Accordingly, the transcriptional, proteomic, and tube formation changes observed following CD109 knockdown supported further investigation *in vivo*, but are not definitive mechanistic evidence of CD109 function during cardiovascular development. Future studies incorporating vascular challenge, hemodynamic stress, cardiovascular injury, or fibrosis, or neonatal regenerative models will be important to determine whether CD109 function becomes more important in these contexts. Collectively, this work establishes CD109 as a marker of coronary endothelial cells and endocardial-derived subpopulations in the valves, and supports a model in which CD109 functions in a context-dependent mannerrather than as an essential regulator of normal cardiovascular development.

## 4 Materials and methods

### 4.1 Animals

All animal experiments were conducted according to NIH guidelines (Guide for the Care and Use of Laboratory Animals) and protocols approved by the MUSC Institutional Animal Care and Use Committee (IACUC) with protocol ID number IACUC-2020–01140. The Tie2-Cre and mWt1/IRES/GFP-Cre (Wt1Cre) mice were described previously (Wessels et al., 2012; Kisanuki et al., 2001). The B6.129(Cg)-Gt (Rosa)26Sortm4 (ACTB-tdTomato,EGFPLuo/J (*Rosa26* ^mT/mG^ dual fluorescence lineage trace reporter expressing membrane bound tdTomato or eGFP) mouse was obtained from the Jackson Laboratory. C57BL/6JGpt-Cd109^em1Cflox^/Gpt (*Cd109* ^fl/fl^) mouse was obtained from GemPharmatech. Endocardial-specific *Cd109* knockout animals were generated by crossing *Tie2* ^Cre^;*Cd109* ^fl/+^ males with *Cd109* ^fl/fl^ females. Genotype distribution from cross did not deviate from expected Mendelian ratios (χ^2^ test, p = 0.439; n = 99), indicating no overt embryonic or perinatal lethality associated with *Cd109* deletion. *Cd109* ^fl/+^ and *Cd109* ^fl/fl^ specimens not carrying the Cre transgene were used as controls. Mice were euthanized by decapitation (embryonic and neonatal stages) or by isoflurane induction followed by cervical dislocation in accordance with NIH guidelines. Embryos were considered day 0.5 at midday on the day of vaginal plug detection, and staging was confirmed upon isolation using distinctive features described in *The House Mouse: Atlas of Embryonic Development* (Theiler, 1989). Combined data for both male and female specimens is shown.

### 4.2 Histology, immunofluorescence, and microscopy

Embryos or postnatal hearts were dissected in 1x Phosphate Buffered Saline (PBS) and fixed in 4% paraformaldehyde (PFA) diluted in PBS for 4 h at room temperature. They were then processed through a series of graded alcohols, cleared in toluene, and embedded in paraffin. Hematoxylin and Eosin (H&E) or immunofluorescence staining was performed on 5um tissue sections. Slides were deparaffinized in xylenes and rehydrated through graded alcohols. Antigen retrieval was performed by pressure cooking slides in citric acid-based antigen unmasking solution (Vector, H3300) for 1 min. Sections were incubated for 30 min with 1% bovine serum albumin (BSA) prior to immunostaining to minimize non-specific binding of primary antibodies. Primary antibodies were used against MF20 (DSHB mf20, 1:100), PECAM1 (Dianova dia-310, 1:25), GFP (Aves Labs gfp-1020, 1:500), and CD109 (R&D Systems AF7717, 1:250). Sections were incubated in primary antibody overnight at 4 °C, washed in 1x PBS, and incubated with secondary antibody for 1 h at room temperature. Serial dilutions were performed to determine optimal antibody concentrations, and secondary only controls were used to confirm signal specificity for the primary antibody. Slides were coverslipped with SlowFade Gold Antifade Reagent with DAPI (Invitrogen). Brightfield images were acquired using Olympus BX40. Fluorescence images were acquired with Zeiss AxioImager II and Leica TCS SP8 microscopes.

### 4.3 Protein isolation and immunoblotting

Protein was harvested from cultured Human Umbilical Vein Endothelial Cells or neonatal murine tissues using 1X RIPA buffer (Thermo, 89900) with 1X Protease Inhibitor Cocktail (Abcam, ab271306), boiled at 95 °C for 5 min with 4X Laemmli Buffer (BioRad, 1610747) with 10% 2-mercaptoethanol, and separated by gel electrophoresis using a BioRad mini blot system with 4%–20% Mini PROTEAN TGX Stain-Free Protein Gels (BioRad, 456–8093). Protein was transferred to a nitrocellulose membrane using a BioRad Trans-Blot Turbo Semi-Dry Transfer System with Trans-Blot Turbo Mini Nitrocellulose Transfer Packs (BioRad, 170-4158). Membranes were washed in Ponceau S total protein stain (Thermo, A40000478) and imaged on a BioRad ChemiDoc MP imaging system for downstream normalization. Membranes were washed in Tris Buffered Saline with 1% Tween-20 (TBST) to remove Ponceau S, then blocked with 5% skim milk diluted in TBST. Membranes were then incubated overnight at 4 °C in primary antibody diluted 1:1000 in blocking buffer (CD109, Cell Signaling 24765). Following three 10-min washes in TBST, membranes were then incubated at room temperature for 1 h in HRP-conjugated secondary antibody diluted 1:7500 in blocking buffer. Following three 10-min washes in TBST, membranes were exposed to Pierce ECL Western blotting Substrate (ThermoFisher, 32209) and imaged on a BioRad ChemiDoc MP imaging system. For relative quantification of protein levels, band signal intensities were determined in Adobe Photoshop, normalized to total protein intensity from Ponceau S from the normalization region indicated on the uncropped blot images included in the Supplementary Material, and compared in GraphPad Prism 9. Proteome Profiler Human Angiogenesis Antibody Arrays (R&D Systems, #ARY007) were conducted according to the manufacturer’s instructions, using 300ug of protein per sample. Normalized array measurements for all detected proteins are included in Supplementary Table S3, along with *p-*values from unpaired two-tailed Student’s t-test, further adjusted for multiple comparisons (FDR Benjamini-Hochberg).

### 4.4 Cell culture and siRNA transfection

Pooled, multiple donor human umbilical vein endothelial cells (HUVECs) (Lonza, Basel, Switzerland) were maintained at 37 °C with 5% CO_2_ in endothelial basal medium 2 (Lonza) supplemented with EGM-2 SingleQuots. HUVECs were transfected using an Amaxa Nucleofection system as described by the manufacturer. Briefly, 1x10^6^ cells were transfected per cuvette, with 2 uM of siRNA targeting human CD109 (Santa Cruz Biotechnology, sc-44950) or non-specific scramble control siRNA (sc-37007). Cells were harvested 72 h after transfection for downstream protein or RNA analyses. The efficiencies of all transfections were confirmed and quantified by Western blot.

### 4.5 RNA-sequencing

Total RNA was isolated from n = 3 scramble control and n = 3 *CD109* siRNA treated HUVEC cultures using the RNeasy Micro Kit (QIAGEN) with RNase free DNAse digestion (QIAGEN) to prevent genomic DNA contamination according to manufacturer’s protocol. RNA integrity and concentration were determined by Bioanalyzer (Agilent 2100) utilizing samples with RINs of >9. Total RNA samples were submitted to a commercial provider (Genewiz-Azenta) for paired-end sequencing at 150bp read length and 20–30 M reads per sample. Sequence data (fastq files) was obtained and processed using Partek Flow software. Pre-alignment quality assessment showed that all samples had high quality scores, with generated data averaging >37 Phred and positional quality consistent throughout the 150bp read length. Alignment was done with an implementation of STAR version 2.7.8a using the hg38 human reference genome. Aligned reads were quantified to Partek E/M annotation model built from hg38 Ensembl Transcripts release 111 with default settings. Differential expression analysis was done with DESeq2 (Love et al., 2014), yielding fold changes and False Discovery Rate (FDR) step up adjusted p-values for pairwise comparison of scramble control and CD109 knockdown cultures (Supplementary Table S1). Gene Ontology functional enrichment analysis was conducted using ToppFun (Chen et al., 2009) (Supplementary Table S2). RNA-sequencing data has been deposited in Gene Expression Omnibus (GEO) with accession number GSE329398.

### 4.6 Tube formation assay

HUVECs maintained as described above were seeded onto growth factor reduced Matrigel-coated 24-well plates at a density of 7x10^4^ cells per well 48 h post-transfection with 2 uM of siRNA targeting human CD109 (Santa Cruz Biotechnology, sc-44950) or non-specific scramble control siRNA (sc-37007) as indicated above. Six wells were seeded for each condition, and tube formation was examined by phase-contrast microscopy at 2, 4, and 6 h after seeding. Three different fields were captured per well, and the characteristics of the HUVECs were analyzed between control and knockdown cultures at 6 h using the WimTube module of the Wimasis Image Analysis software (Wimasis, 2016).

### 4.7 Proliferation assay

HUVECs were transfected with scramble control or CD109 siRNA as described above. 24 h post transfection, cells were seeded in 24-well plates at low density (5,000 cells per well). Four wells per condition were analyzed at 24-h intervals over a 4 day period. Culture medium was removed, and cells were washed with PBS, followed by staining with 0.2% crystal violet solution. Excess stain was removed by washing with distilled water, and plates were allowed to dry. Crystal Violet was then solubilized using a 1% SDS solution, and absorbance was measured at 550 nm using a spectrophotometer. Negative control wells were processed in parallel that contained no cells. Absorbance values were compared between conditions at each timepoint.

### 4.8 Histological quantifications

To quantify the lineage origin of CD109^+^ cells within the developing mitral valve, E17 *Wt1* ^Cre^;*Rosa26* ^mT/mG^ and *Tie2* ^Cre^;*Rosa26* ^mT/mG^ embryos were immunolabeled for CD109, GFP, and MF20 and stained with DAPI. Three histological sections spanning the mitral valve leaflets were analyzed from each specimen (n = 3 embryos per lineage trace model). Nuclei of CD109^+^ cells within the parietal leaflet were counted and analyzed for co-localization with GFP lineage reporter expression. The percentage of CD109^+^ cells co-labeled with the lineage reporter was calculated for each section and averaged across sections to generate a single biological replicate value for each embryo. Mitral valve morphometrics were analyzed on H&E stained sections spanning the entire mitral valve, and volume statistics were calculated in AMIRA 3D software. For compact myocardium thickness and vascular invasion measurements, the measure tool within the CellSens Standard imaging software was used. Four sections per specimen were analyzed, and six measurements per section were taken. Measurements for each specimen were averaged to generate a single value for each biological replicate. Vascular density quantification was performed using CellProfiler 3.1.8 software. Cropped images of left ventricular compact myocardium were analyzed to identify and measure DAPI^+^ nuclear area and PECAM1^+^ vasculature area. Four sections per specimen were analyzed, and measurements for each specimen were averaged to generate a single value for each biological replicate. Data points shown in graphical analyses represent average values from individual specimens.

### 4.9 Statistics

All quantitative data are presented as mean ± standard deviation. Statistical analyses were performed using GraphPad Prism 9. For comparison of valve volumes, vasculature, and myocardial thickness between control and *Tie2* ^Cre^;*Cd109* ^fl/fl^ groups at individual developmental stages, two-tailed unpaired Student’s t-tests were used to compare means. Analyses were performed independently for each developmental timepoint and measurment examined.

Biological replicates (n) represent individual embryos or animals unless otherwise specified. Data distributions were visually inspected for gross deviations from normality prior to parametric testing. Biological n numbers and exact *p*-values are reported in the corresponding figure legends. No formal correction for multiple comparisons was applied, as analyses were limited to predefined morphological assessments selected prior to experimentation.

## Data Availability

The datasets presented in this study can be found in online repositories. The names of the repository/repositories and accession number(s) can be found in the article/Supplementary Material.
