# Supplementary figures and images for: CD109 exhibits a dynamic expression pattern in coronary endothelium and endocardial-derived valve mesenchyme during heart development with preserved morphogenesis following endothelial-specific deletion
Source: Front Cell Dev Biol. 2026 Jun 19;14:1867997. doi: 10.3389/fcell.2026.1867997 (PMC13328117; doi:10.3389/fcell.2026.1867997)

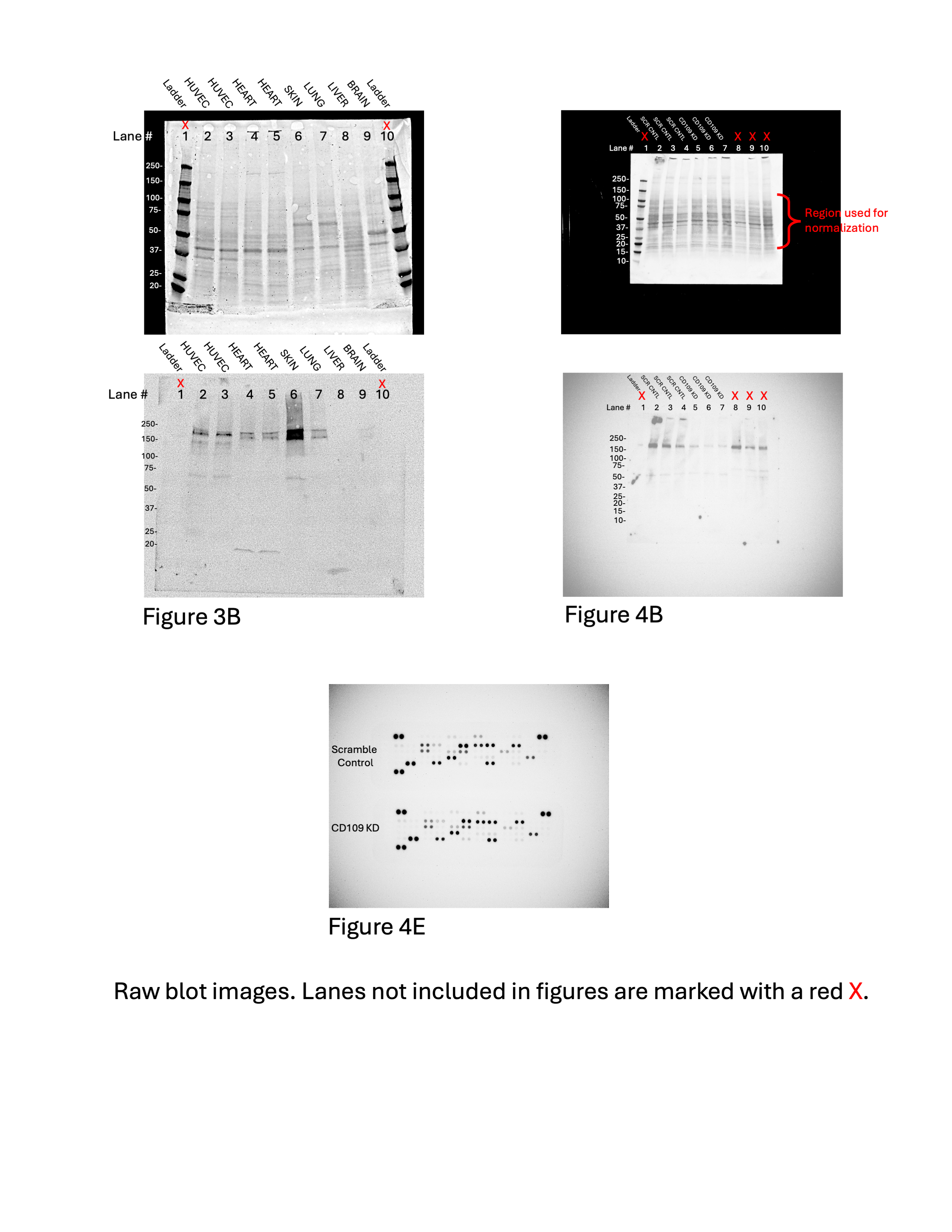

Supplement: Supplementary file 1 [file Image1.tiff]
